# Intraoperative use of remifentanil and opioid induced hyperalgesia/acute opioid tolerance: systematic review

**DOI:** 10.3389/fphar.2014.00108

**Published:** 2014-05-08

**Authors:** Sang Hun Kim, Nicoleta Stoicea, Suren Soghomonyan, Sergio D. Bergese

**Affiliations:** ^1^Department of Anesthesiology and Pain Medicine, School of Medicine, Chosun UniversityGwangju, South Korea; ^2^Department of Anesthesiology, Ohio State University Wexner Medical CenterColumbus, OH, USA; ^3^Department of Neurological Surgery, Ohio State University Wexner Medical CenterColumbus, OH, USA

**Keywords:** remifentanil, opioid-induced hyperalgesia, opioid tolerance, intraoperative, postoperative

## Abstract

**Introduction:** The use of opioids has been increasing in operating room and intensive care unit to provide perioperative analgesia as well as stable hemodynamics. However, many authors have suggested that the use of opioids is associated with the expression of acute opioid tolerance (AOT) and opioid-induced hyperalgesia (OIH) in experimental studies and clinical observations in dose and/or time dependent exposure even when used within the clinically accepted doses. Recently, remifentanil has been used for pain management during anesthesia as well as in the intensive care units because of its rapid onset and offset.

**Objectives:** Search of the available literature to assess remifentanil AOT and OIH based on available published data.

**Methods:** We reviewed articles analyzing remifentanil AOT and OIH, and focused our literature search on evidence based information. Experimental and clinical studies were identified using electronic searches of Medline (PubMed, Ovid, Springer, and Elsevier, ClinicalKey).

**Results:** Our results showed that the development of remifentanil AOT and OIH is a clinically significant phenomenon requiring further research.

**Discussions and Conclusions:** AOT – defined as an increase in the required opioid dose to maintain adequate analgesia, and OIH – defined as decreased pain threshold after chronic opioid treatment, should be suspected with any unexplained pain report unassociated with the disease progression. The clinical significance of these findings was evaluated taking into account multiple methodological issues including the dose and duration of opioids administration, the different infusion mode, the co-administrated anesthetic drug’s effect, method assessing pain sensitivity, and the repetitive and potentially tissue damaging nature of the stimuli used to determine the threshold during opioid infusion. Future studies need to investigate the contribution of remifentanil induced hyperalgesia to chronic pain and the role of pharmacological modulation to reverse this process.

## INTRODUCTION

Even though remifentanil increases analgesia and respiratory depression in a dose-dependent manner (Hughes et al., 1992; Egan et al., 1993; Glass et al., 1993; Westmoreland et al., 1993; Kapila et al., 1995), these effects disappear rapidly after discontinuing administration of the drug because of the extremely short elimination half-life (9.5 ± 4 min). Especially, remifentanil has the shortest context-sensitive half-time and terminal elimination half-life among other opioid after 3-h infusion (Kapila et al., 1995). Therefore, remifentanil can be given in high doses throughout surgery without the risk of delayed postoperative recovery or respiratory depression. Because of its pharmacodynamic and pharmacokinetic effects, remifentanil has been used in clinical anesthesia as an induction and maintenance agent, and postoperative pain management in the intensive care units.

Most of the studies conducted with remifentanil showed cardiovascular responses during perioperative manipulations. They recommended a bolus injection of remifentanil of 1 μg/kg as more effective dose in reducing the pressor response during laryngoscopy and tracheal intubation (McAtamney et al., 1998; O’Hare et al., 1999). However, while the cardiovascular responses reaches a peak 1–2 min after laryngoscopy and intubation, and usually subsides within 5–6 min (Singh et al., 1995), the context-sensitive half-time of bolus remifentanil is only 3.2 min (Glass et al., 1993; Kapila et al., 1995). Therefore, remifentanil bolus alone is not enough to attenuate the responses and the use of a bolus-infusion regimen is required (McAtamney et al., 1998). The commonly accepted and recommended dose of remifentanil is 1 μg/kg followed by an infusion of 0.5–1 μg/kg/min for induction of anesthesia or 0.05–2.0 μg/kg/min for maintenance of anesthesia (Burkle et al., 1996; Hall et al., 2000; Sneyd et al., 2001). In postoperative period, remifentanil continuous infusion (CI) also can be used for controlling the pain, and the final remifentanil infusion rates have been reported as 0.05–0.26 μg/kg/min for satisfactory analgesia after surgery (Bowdle et al., 1996, 1997; Schuttler et al., 1997; Yarmush et al., 1997; Sneyd et al., 2001).

Common concerns regarding the use of opioids are potential detrimental side effects, physical dependence, and addiction. However, an additional concern has recently risen that these opioids can induce an acute tolerance and hyperalgesia in dose and/or time dependent manner even when used within clinical accepted doses. They provide straight analgesic and antihyperalgesic effects originally, but subsequently are associated with expression of hyperalgesia (Angst and Clark, 2006). The use of opioids may seem to be a double-edged sword. In other words, patients receiving opioids to control their pain somewhat paradoxically may become more sensitive to pain as a direct result of opioid therapy.

Therefore, a review of literature was carried out to analyze acute tolerance and/or hyperalgesia induced by remifentanil in perioperative period using electronic searches of Medline (PubMed, Ovid, Springer, and Elsevier, ClinicalKey). The objective was to address the following issues: (1) what is the definition of acute opioid tolerance (AOT) and opioid-induced hyperalgesia (OIH)? (2) Dose remifentanil may induce the acute tolerance and hyperalgesia? (3) Are AOT and OIH significant enough to consider reducing the dose of remifentanil or adopting preventive modulations?

## DEFINITION OF ACUTE OPIOID TOLERANCE AND OPIOID-INDUCED HYPERALGESIA

Before we discuss AOT and OIH induced by remifentanil, we have to understand the definitions of AOT and OIH because management for pain control is different according to make a diagnosis one of them; While AOT can be overcome by increasing the dosage, OIH even worsens the pain. Consequently, OIH is reduced by reducing or eliminating the opioid.

Opioid-induced hyperalgesia is defined as a state of nociceptive sensitization, which is characterized by a paradoxical response, whereby a patient receiving opioids for pain treatment might have an increased sensitivity to painful stimuli (Angst and Clark, 2006; Chu et al., 2008). Even though controlled preclinical experiments have defined OIH in animals as a decrease in pain threshold from baseline after chronic administration of opioids (Fishbain et al., 2009), there still is no accepted operational definition of OIH among researchers in human clinical trials; hyperalgesia is defined as decrease in either pain threshold or pain tolerance after chronic opioid exposure (Martinez and Fletcher, 2012). Pain threshold is the lowest intensity of stimulation at which pain is experienced, and pain tolerance is the amount of pain from a given stimulus a person can handle before seeking relief.

Opioid-induced hyperalgesia often is confused with opioid tolerance because of the manifestations of similar symptoms. AOT is defined as an increase in the dose required maintaining adequate analgesia in patients receiving opioid medication for the treatment of pain in clinical settings (Angst and Clark, 2006; Chu et al., 2008). Clinical data indicates that early postoperative pain scores and subsequent greater demand of opioids could be attributed to tolerance, while the greater requirement for opioids at a later recovery stage could be associated with OIH after high-dose remifentanil anesthesia.

## GENERAL CONSENSUS OF AOT AND OIH

Recently, many studies have focused on the development of AOT and OIH after using opioids based on idea that OIH might be a potential risk factor for the development of chronic pain after surgery (Perkins and Kehlet, 2000; Woolf and Salter, 2000; Wilder-Smith and Arendt-Nielsen, 2006). The circumstances under which OIH may occur are also not yet entirely understood but may include high doses, long-term treatment, or abrupt changes in concentrations (Simonnet and Rivat, 2003). Most authors have suggested that the higher dose of opioids induce the higher tolerance and/or hyperalgesia. OIH was observed either to follow analgesia and lasted long after opioid exposure ended (Celerier et al., 2000; Kissin et al., 2000; Li et al., 2001), or during continuous opioid exposure (Laulin et al., 1999; Vanderah et al., 2000). It has been reported that high intraoperative doses of opioids not only increase postoperative pain scores and acute morphine consumption, but also induce significant nociceptive threshold changes (Chia et al., 1999; Guignard et al., 2000; Mao, 2002; Angst et al., 2003; Joly et al., 2005; Van Elstraete et al., 2005; Cabanero et al., 2009). Celerier et al. (2000) showed that fentanyl injection was associated with sustained lowering of the nociceptive threshold below baseline value, and the higher the fentanyl dose used, the more pronounced was the fentanyl-induced hyperalgesia. Laulin et al. (1999) reported that repeated once-daily heroin injections induced a gradual lowering of the nociceptive threshold which progressively masked a sustained heroin analgesic functional effect, which is suggested as opiate tolerance.

If all types of opioids have been shown to induce such a dose-dependent hypersensitivity, exposure to short-acting opioids, such as remifentanil, seems more likely to be responsible for postoperative high pain scores, high morphine consumption, and hypersensitivity to pain (Guignard et al., 2000; Angst et al., 2003; Hood et al., 2003; Koppert et al., 2003a). A relatively large-dose of intraoperative remifentanil has been shown to induce postoperative hyperalgesia more rapidly and more frequently as compared with longer-acting opioids (Derrode et al., 2003; Joly et al., 2005; Koppert and Schmelz, 2007). These results are in agreement with the clinical observation of increased postoperative pain and morphine requirement (Zarate et al., 1999; Guignard et al., 2000, 2002).

As many papers suggest, remifentanil could induce AOT and/or OIH (Vinik and Kissin, 1998; Hayashida et al., 2003; Gomez de Segura et al., 2009; Aguado et al., 2011). Many authors documented that remifentanil CI alone induced AOT in the dose-dependent manner within first few hours in animal models (Hayashida et al., 2003; Aguado et al., 2011). A study in healthy human volunteers performed by Vinik and Kissin (1998) also suggested that AOT was profound and developed very rapidly, and remifentanil of 0.1 μg/kg/min resulted in the maximum analgesic effect in 60–90 min, and then began to decline despite the constant-rate infusion, eventually reaching 1/4 of the peak value after 3 h of infusion, measuring by cold thermal and mechanical noxious stimulation (Vinik and Kissin, 1998).

A small number of clinical studies have looked at AOT and OIH in the setting of acute perioperative opioid exposure. A series of studies in patients undergoing surgery suggested that exposure to a high rather than to a low intraoperative opioid dose was associated with the increased opioid consumption and/or pain in the postoperative period (Cooper et al., 1997; Chia et al., 1999; Guignard et al., 2000; Joly et al., 2005). A feasible explanation for these findings is either the development of acute tolerance on the rescue opioids for controlling the postoperative pain or a possible OIH in patients exposed to a high intraoperative opioid dose (Angst and Clark, 2006). Guignard et al. (2000) suggested that AOT as well as OIH might be induced by the acute exposure to large doses of opioids. The patients received 0.3 ± 0.2 μg/kg/min of the intraoperative remifentanil required morphine significantly earlier and needed nearly twice more morphine in the first 24 postoperative hours than those who remifentanil was kept constant at 0.1 μg/kg/min. Furthermore, despite higher morphine requirement, higher pain scores were observed in group that remifentanil was infused higher. Joly et al. (2005), in their randomized, double-blind study, showed that intraoperative remifentanil of 0.4 μg/kg/min triggered the larger hyperalgesia as well as the larger morphine consumption for 48 postoperative hours, compared with remifentanil of 0.05 μg/kg/min. In prospective, randomized, double-blind study, they suggested that remifentanil of mean 0.28 μg/kg/min was associated with the development of clinically relevant AOT, in patients underwent the general anesthesia using propofol infusion (Crawford et al., 2006).

Many authors showed that the OIH can develop differently for different types of pain such as transdermal electrical stimulation (Angst et al., 2003; Hood et al., 2003; Koppert et al., 2003a,b; Troster et al., 2006), cold pressor pain (Compton et al., 2003, 2004), and pressure-evoked pain (Luginbuhl et al., 2003). Transdermal electrical stimulation used for inducing mechanical hyperalgesia on an experimental skin lesion rendered hyperalgesia in human volunteers. These investigators suggested that 30–90 min of remifentanil exposure significantly enlarge the skin area with pre-existing mechanical hyperalgesia by transdermal electrical stimulation with direct relation of the infusion duration and the opioid dose (Angst et al., 2003; Hood et al., 2003; Koppert et al., 2003a,b; Troster et al., 2006). This hyperalgesia was sustained up to 4 h after stopping infusion (Hood et al., 2003). They also documented that the pain score was increased and the pain threshold was decrease, by dose-dependent manner, after the discontinuation of opioids (Angst et al., 2003; Hood et al., 2003; Koppert et al., 2003a). In a model of acute physical opioid dependence, which withdrawal was precipitated with the opioid antagonist naloxone after a single injection of opioid (Compton et al., 2003, 2004), the authors showed that sensitivity to cold pressor pain was significantly increased after injection of naloxone and OIH may be triggered if opioid effect is suddenly disappeared or reversed. Finally, Luginbuhl et al. (2003) suggested that remifentanil alone induced significant hyperalgesia detected by test for pressure pain tolerance threshold in volunteers.

## SUGGESTED DOSES OF REMIFENTANIL INDUCING AOT AND OIH

Most articles documented that AOT and OIH were induced during and after remifentanil infusion at ≥0.1 μg/kg/min (Vinik and Kissin, 1998; Glass et al., 1999; Guignard et al., 2000; Gustorff et al., 2002; Angst et al., 2003; Koppert et al., 2003a; Joly et al., 2005; Angst et al., 2009; Cabanero et al., 2009). In a mouse model, they suggested that remifentanil-induced hyperalgesia was induced by dose-dependent manner and the calculated ED50s for thermal and mechanical hyperalgesia was 1.7 (95% confidence interval, 1.3–2.1) and 1.26 (1.0–1.6) μg/kg/min, respectively (Cabanero et al., 2009). Infusion of remifentanil at a rate of 0.1 μg/kg/min induced the very rapid and profound acute tolerance during infusion (Vinik and Kissin, 1998; Glass et al., 1999), while a rate of 0.08 μg/kg/min could not induce acute tolerance in the cold pressor test and in models of electrical and heat pain (Gustorff et al., 2002). Angst et al. (2003) showed that pre-existing mechanical hyperalgesia was significantly aggravated after discontinuation of remifentanil at a rate of 0.10 μg/kg/min. Koppert et al. (2003a) also suggested that even though remifentanil dose-dependently reduced pain and mechanical hyperalgesia during the infusion, after discontinuation of infusion, pain and mechanical hyperalgesia increased significantly in group which was administered remifentanil at a rate of 0.10 μg/kg/min compared with at a rate of 0.05 μg/kg/min. Furthermore, in the patients receiving higher intraoperative remifentanil, such as 0.3 ± 0.2 and 0.4 μg/kg/min, than 0.10 μg/kg/min, the time of first morphine requirement was significantly earlier and the morphine consumption in the first 24 or 48 postoperative hour was significantly lager, which suggest that remifentanil causes AOT and hyperalgesia (Guignard et al., 2000; Joly et al., 2005). Therefore, AOT as well as OIH might be induced by the acute exposure to large doses of opioids.

In studies on AOT and OIH, while most authors have used the CI mode, there are a few reports using the target-controlled infusion (TCI) mode (Hood et al., 2003; Angst et al., 2009; Shin et al., 2010; Richebe et al., 2011). Hood et al. (2003) showed that areas of hyperalgesia continuously enlarged 4 h after remifentanil (targeted concentration of 3.1 ± 1.2 ng/ml) was stopped, to 180% ± 47%. Shin et al. (2010) suggested that remifentanil using TCI at 4 ng/ml induced the more increased cumulative morphine consumption and postoperative hyperalgesia than that at 1 ng/ml during sevoflurane anesthesia. This result is similar with that of previous studies using CI mode; higher dose of opioid, higher development of AOT and OIH. The infusion rate for remifentanil 0.1 μg/kg/min can achieve a stable plasma concentration ranging between 2.7 and 2.9 ng/ml during the infusion (Angst et al., 2003). A bolus of remifentanil 1 μg/kg followed by infusion 0.2 μg/kg/min will produce stable plasma concentrations of 4–5 ng/ml within a few minutes (Minto et al., 1997). Angst et al. (2009) documented that target remifentanil concentrations corresponding to infusion rates of 0.065 and 1.3 μg/kg/min did not induce tolerance in any of their pain models. Remifentanil concentrations of 1.6 and 3.2 ng/ml, which are commonly used in a clinical setting to provide analgesia during surgery, correspond to steady-state concentrations achieved when infusing remifentanil at a constant rate of about 0.065 and 0.13 μg/kg/min (Glass et al., 1999). Therefore, according these references, ≥0.1 μg/kg/min using CI mode and ≥2.7 ng/ml using TCI mode seem to be sufficient to develop hyperalgesia.

## CONTROVERSIAL RESULTS ON DEVELOPMENT OF AOT AND OIH BY REMIFENTANIL

There is controversial results on AOT and OIH induced by remifentanil at the perioperative period, even though many authors suggested that remifentanil could induce them (Schraag et al., 1999; Cortinez et al., 2001; Hansen et al., 2005; Lahtinen et al., 2008; Angst et al., 2009; Yeom et al., 2012). Angst et al. (2009) documented that 3 h infusion of remifentanil of up to 4.0 ng/ml was not associated with the development of significant tolerance to analgesic in placebo-controlled, double-blind study. Cortinez et al. (2001) also suggested that remifentanil-based anesthesia (0.23 ± 0.10 μg/kg/min, average duration; 116 min) did not induce the AOT when compared with sevoflurane-based anesthesia in patients undergoing elective gynecologic surgery. Hansen et al. (2005) investigated how remifentanil of 0.4 μg/kg/min intra-operatively affected post-operative pain and opioid consumption after major abdominal surgery. In a double-blind study, although they found higher visual analog scale score in the remifentanil group compared with placebo during the immediate postoperative period, this difference was no longer significant 2 h after surgery or during the remainder of the 24-h observation period. A prospective, randomized, double-blind study, showed that 3 h infusion of remifentanil of 0.3 μg/kg/min did not increase postoperative pain or opioid consumption in cardiac surgery patients who underwent sufentanil/propofol-based general anesthesia (Lahtinen et al., 2008). Yeom et al. (2012) suggested that remifentanil did not appear to cause AOT and OIH in patients undergoing spinal fusion even though there was significant differences in the mean intraoperative infusion rate of remifentanil (0.16 μg/kg/min vs. 0.03 μg/kg/min) for a short period of time (averaging 225 and 216 min, respectively). Schraag et al. (1999) found that Remifentanil infusion using specially designed patient-maintained TCI systems, which was controlled according to the patients demand for postoperative pain control by up-and-down method (intervals 0.2 ng/ml) did not show any evidence of rapid development of acute tolerance in patients maintained hypnotic dose of remifentanil in the first six postoperative hours from end of total intravenous anesthesia with remifentanil and propofol.

## FACTORS THAT LEAD TO DISCREPANCIES REGARDING AOT AND OIH

The clinical relevance of above mentioned results is questionable and there is some limitation in negative results concerning AOT and OIH. These discrepancies can be explained by multiple methodological issues including the administrated dose and duration of opioids administration, the different infusion mode, the co-administrated anesthetic drug’s effect, method assessing pain sensitivity, and the repetitive and potentially tissue damaging nature of the stimuli used to determine the threshold during opioid infusion (Angst and Clark, 2006; Lahtinen et al., 2008; Fishbain et al., 2009; Simonnet, 2009).

First, we can explain these discrepant results by differences in exposed opioid doses and administration duration. Studies reporting positive results have shown that AOT develops in dose-dependent fashion and only becomes evident when total opioid exposure is quite high. The non-significant increase of postoperative pain and opioid consumption in studies reporting negative results may be noticed because of lower total intraoperative opioid exposure when compared with the positive results, suggesting a dose-dependent effect of opioids on the development of OIH (Guignard et al., 2000; Cortinez et al., 2001; Lee et al., 2005). Cabanero et al. (2009) agreed that remifentanil induced dose-dependent pronociceptive effects for thermal and mechanical hyperalgesia, which lasted longer with higher doses, but they suggested that the duration of infusion did not alter the pronociceptive effects of remifentanil. This negative result might be explained by the shorter exposed duration, just over 30 or 60 min, than the positive results studies, in which Acute tolerance is typically investigated during CI over 2–3 h, whereas hyperalgesia is usually assessed within 1 h post-infusion (Guignard et al., 2000, 2002; Laulin et al., 2002; Hayashida et al., 2003; Gomez de Segura et al., 2009; Benito et al., 2010; Aguado et al., 2011; Ishida et al., 2012; Ishii et al., 2013). In the recent animal study, they showed that intravenous remifentanil infusion alone induced transient hyperalgesia associated with the duration of exposure to remifentanil (Ishida et al., 2012). While 30 min remifentanil infusion did not induce hyperalgesia, 120 min remifentanil infusion induced the hyperalgesia regardless of dose. However, hyperalgesia was not sustained more than 60 min. Other study also documented that remifentanil-induced hyperalgesia started from 2 h after surgery and reached its peak at 24–48 h after surgery (Gu et al., 2009).

Also, it is not sufficient to explain the cause of these discrepancies by using the dose and duration of remifentanil infusion. Some authors have reported the negative results even though they used remifentanil infusion rate that was enough to develop hyperalgesia. It can be partially explained by the effect of co-administrated anesthetic drugs, such as propofol, sevoflurane and nitrous oxide. Fodale et al. (2006) suggested that AOT was not induced when remifentanil was co-administered with propofol or sevoflurane, which produced an inhibiting effect at NMDA receptors neutralizing the remifentanil stimulation on these receptors. However, Solt et al. (2006) documented that sevoflurane has only a minimal inhibitory effect on NMDA receptors, which are considered to be involved in the development of opioid-related hypersensitivity (Tompkins and Campbell, 2011). Relatively low sevoflurane concentrations (1.0%) reverse OIH, but there was the lack of effect of sevoflurane concentrations of 1.0 and 1.5% to oppose hyperalgesia following high-dose opioids (Solt et al., 2006; Richebe et al., 2009). Shin et al. (2010) also suggested that remifentanil-induced hyperalgesia was not apparent during propofol anesthesia compared with the effect produced during sevoflurane anesthesia even though dosage of remifentanil was increased from 1.0 to 4.0 ng/ml. This result can be supported by that propofol may have some modulatory effect on OIH, through inhibition of the NMDA subtype of the glutamate receptor (Orser et al., 1995; Kingston et al., 2006), which is one of the potential mechanisms that induced the OIH, and possibly through interactions with gamma-aminobutyric acid (GABA-A) receptors at the supraspinal level (Wang et al., 2004; Singler et al., 2007). Specifically, sub-hypnotic dose of propofol has analgesic effects, which delayed the onset of anti-analgesia after remifentanil infusion (Singler et al., 2007). However, the clinical significance of these findings, especially in higher dosages used in the intraoperative setting, remains to be studied. In addition, some authors ignored the impact that nitrous oxide might have against the AOT and hyperalgesia (Cortinez et al., 2001; Yeom et al., 2012). Echevarria et al. (2011) reported that the group using the 70% nitrous oxide with remifentanil of 0.3 μg/kg/min showed a greater decreased mechanical threshold than the group without nitrous oxide at postoperative 12–18 h, even though the postoperative pain scores and cumulative morphine consumption was similar between the groups. Lee et al. (2005) suggested that 70% nitrous oxide showed comparable effect on the postoperative opioid consumption similar to remifentanil at mean 0.17 μg/kg/min.

Next, different infusion mode also can influence the development of AOT and OIH even though they have an equipotential effect on the pain control. TCI has been shown not only to improve intraoperative hemodynamic stability but also to decrease intraoperative remifentanil requirements (Minto et al., 1997; De Castro et al., 2003). Interestingly, Richebe et al. (2011) evaluated whether the use of TCI mode also would lead to decrease in early postoperative period hyperalgesia after cardiac surgery. They suggested that an infusion of intraoperative remifentanil using TCI mode (7 ng/ml) reduced postoperative hyperalgesia, compared with that using CI mode (0.3 μg/kg/min), and this decrease in postoperative hyperalgesia was sustained and lasted throughout the 1st postoperative week. The conclusion of the study was supported by the difference of intraoperative infused total remifentanil dose, which was greater in CI than in TCI group.

Then, the possibility of cumulative tissue injury manifesting as AOT or OIH should be carefully excluded (Petrenko et al., 2012). Especially, this is likely to occur with repetitive testing protocols in a situation in which protective withdrawal reflexes are impaired or abolished by opioid administration. In Luginbuhl et al.’s (2003) study, they exposed volunteers to significantly higher nociceptive input during remifentanil vs. during saline placebo administration. It cannot be excluded that postinfusion hyperalgesia resulted from more intense noxious stimulation during the remifentanil infusion rather than the opioid administration itself. On the other hand, Ishii et al. (2013) documented that neither acute tolerance nor hyperalgesia was observed even in the setting when they used a tapered remifentanil infusion to rapidly attain maximum analgesic effect of remifentanil and tried to minimize the repetitive and potentially tissue damaging nature of the stimuli.

Finally, it might contribute to influence of development of OIH, in studies exposing patients to high intraoperative opioid doses, whether assessment of pain sensitivity before and after surgery was formally done or not. No causal relationship between acute perioperative opioid exposure and development of OIH can be established without direct measurement of pain sensitivity. If patients have a comorbidity affecting sensory thresholds preoperatively, this condition could distort postoperative measures on OIH. The German Network on Neuropathic Pain established a standardized quantitative sensory testing (QST) protocol to investigate the somatosensory thresholds in healthy subjects and in patients with neuropathic pain (Rolke et al., 2006). Reference values from healthy subjects could be used to establish normal sensory functioning in patients before anesthesia. In a clinical setting, this direct measure could be used for distinguishing between OIH and AOT, because of clinical importance, as AOT can be overcome by dose increase, while OIH may be aggravated by the same intervention. Without direct measures to assess hyperalgesia such as QST, the results are not easy to distinguish from acute tolerance (Vinik and Kissin, 1998). Clinical studies examining remifentanil-induced hyperalgesia by QST showed that, at a relatively high dose, remifentanil decreased the pain threshold (Gustorff et al., 2002; Joly et al., 2005; Schmidt et al., 2007; Song et al., 2011; Yalcin et al., 2012). These studies used remifentanil infusions at clinically standard rates, and all of them showed clear hyperalgesia either shortly after discontinuing infusion or 1 and 2 days postoperatively. However, most studies confirmed the reality of this phenomenon using indirect evidences, such as greater postoperative pain and morphine consumptions instead of using QST (Guignard et al., 2000; Rauf et al., 2005). Treskatsch et al. (2014) did not demonstrate any influence on clinical outcome parameters of pain after remifentanil of >0.2 μg/kg/min, and they suggested that indirect evidences might be less sensitive to detect OIH compared with QST. Therefore, we think that a further study is needed to reveal the development of AOT and OIH using QST.

## IMPORTANCE OF AOT AND/OR OIH IN CONSIDERING THE REDUCTION OF REMIFENTANIL DOSAGE AND ADOPTING PREVENTIVE MODULATIONS

Forty-one percentage of all surgical patients still experience moderate to severe acute postoperative pain and that 24% experience inadequate pain relief (Dolin et al., 2002). It has recently been highlighted that chronic pain as a direct result of surgery is more common than previously recognized (Perkins and Kehlet, 2000). Furthermore, in cohort study on postoperative remifentanil-induced hyperalgesia, its incidence was reported 16.1% of patients undergoing general anesthesia with remifentanil (Ma et al., 2011). Hyperalgesia and increased pain in the postoperative period is now considered a major candidate mechanism for the development of chronic pain (Perkins and Kehlet, 2000; Woolf and Salter, 2000; Wilder-Smith and Arendt-Nielsen, 2006). Therefore, less postoperative hyperalgesia results in better acute postoperative pain control (Joly et al., 2005), and interventions associated with alterations of postoperative hyperalgesia are also associated with changes in acute postoperative pain outcomes (Guignard et al., 2000; Rauf et al., 2005).

However, there is a lack of good quality clinical research in this area, despite the fairly extensive basic science evidence. In a structured evidence-based review for all levels of evidence on OIH in humans (Fishbain et al., 2009), they suggested that there was not sufficient evidence to support or refute the existence of OIH in humans except in the case of normal volunteers receiving opioid infusions. There was consistent evidence that opioid infusion in normal volunteers induced either an increase in secondary hyperalgesia or allodynia, and there was inconsistent evidence on pain threshold and tolerance in normal volunteers or chronic pain patients although the threshold decreased with opioid infusion. They also documented that using of opioid in perioperative period increased the postoperative pain or opioid requirements with inconsistent evidence. In other recent evidence based systematic review (Rivosecchi et al., 2014), they suggested that remifentanil might induce a degree of hyperalgesia, but it did not reach a level of clinical significance that requires prevention.

There are some questions regarding the importance of the drug use to prevent AOT and OIH in postoperative patients and whether the assessment of OIH at immediate postoperative period is suitable. Clinical studies assessing the preventive effect of drugs on OIH in the immediate postoperative period, showed that the clinical benefit is either absent (Echevarria et al., 2011), limited to a moderate opioid-sparing effect (Joly et al., 2005; Shin et al., 2010), or a slight reduction in pain scores (Shin et al., 2010; Song et al., 2011). According to these results, Martinez and Fletcher (2012) suggested that the immediate postoperative period may not be the optimal period to detect the preventive effects on OIH, even though additional clinical data need to confirm it.

Furthermore, Simonnet and Rivat (2003) suggested that OIH should be considered as a normal adaptive response counteracting the perturbations caused by administration of analgesic opioids. However, it is tempting to speculate that the long lasting hyperalgesia induced by endogenous or exogenous opioids may still facilitate learning processes and memorization of drives so that environmental changes which might lead to pain may be better avoided. From a medical viewpoint, OIH following a first opioid administration is not a passive response but it might be considered as the first step of an active process leading to pain sensitization. This suggests that opioids have reinforced a nociceptive memory which could contribute to pain chronicization.

## CONCLUSION

Current experimental and clinical data generally support the development of AOT and OIH in specific settings such as acute remifentanil exposure in human volunteer and postsurgical pain cohorts when remifentanil was infused at ≥0.1 μg/kg/min either alone or with inhalation anesthetics. Therefore, in these situations, clinicians need to be cautious for the possibility of the development of AOT and OIH, which may impair treatment of pain or even aggravate preexisting pain. Clinicians should suspect manifestation of OIH when opioid treatment effect seems to decline in the absence of disease progression, with unexplained pain reports or allodynia unassociated with the site of injury. According to the previous reported results, co-administrated anesthetic drugs, such as propofol and nitrous oxide, and using of TCI model seem to be helpful to modulate the development of the AOT and OIH. However, there are not sufficient data to support the evidence of modulatory effect of them. Finally, we also cannot find any strong consistent evidence to support the need to reduce the dose of remifentanil or apply the modalities for preventing the AOT and the OIH. Consequently, further studies will need to investigate whether remifentanil induce the AOT and the OIH after general anesthesia using propofol, nitrous oxide, or TCI mode through high quality prospective trials. And the development of the AOT and the OIH should be evaluated with direct measures such as QST. It is also important to investigate if remifentanil-induced hyperalgesia may contribute to the development of chronic pain, and if this contribution can be attenuated or even reversed through pharmacologic modulation.

## Conflict of Interest Statement

The authors declare that the research was conducted in the absence of any commercial or financial relationships that could be construed as a potential conflict of interest.
